# A crop recommendation using improved expert-guided constraint aware differential evolution and CatBoost surrogate learning

**DOI:** 10.3389/fpls.2026.1884580

**Published:** 2026-07-21

**Authors:** Pavithra Mahesh, Rajkumar Soundrapandiyan

**Affiliations:** School of Computer Science and Engineering, Vellore Institute of Technology, Vellore, Tamil Nadu, India

**Keywords:** CatBoost, crop recommendation, differential evolution, multi-criteria, optimization, scenario aware, surrogate model

## Abstract

Planning for sustainable crops, among other things, depends on smart decision-support systems capable of managing multi-criteria interactions and different environmental conditions. This study presents a multi-criterion, scenario-aware crop recommendation system developed by coupling Improved ECA-DE (Expert-guided Constraint-Aware Differential Evolution) optimization with CatBoost (Categorical Boosting) surrogate learning. The system assesses the suitability of crops using a range of agro-environmental factors, such as soil type, soil pH, temperature, humidity, water requirement, crop duration, and water source under three scenarios: balanced, drought, and short-season conditions. Improved ECA-DE is used for the determination of criterion weights, and the CatBoost model approximates expert-based suitability scores very effectively. The prediction performance of the proposed method was very high, with coefficient of determination (*R*^2^) = 0.9975, Mean Absolute Error (MAE) = 0.0043, and Root Mean Square Error (RMSE) = 0.0056, showing almost perfect matches between the predictions and expert judgments. In addition, the Improved ECA-DE can drastically lower the computation time and memory usage while providing Standard DE matching in terms of optimization quality. In addition, the scenario-based assessment revealed that the method can accurately represent the trade-offs in suitability, water requirement, and crop duration, thus facilitating adaptive recommendations under different conditions. The proposed framework is a precise, interpretable, and resource-efficient tool for smart and sustainable agricultural decision-making.

## 1 Introduction

The decision-making process in agriculture is gradually moving away from solely relying on intuition for crop selection to using data-driven planning that can take into account local soil conditions, limitations of resources, and weather fluctuations (Zhai et al., 2020; Shamshiri et al., 2024; Saha et al., 2025). Such in smart agriculture, recommendation systems are expected not only to determine the ones that are crop suitable, but also to provide at least support for the production and planning decision intervals consistent with changing scenarios (Alam et al., 2025; Mandal et al., 2024). Recent surveys on smart agriculture, Artificial Intelligence of Things (AIoT), and digital agriculture indicate that intelligent systems are being used for operational planning, resource management, and adaptive decision support in a broader way in addition to only prediction tasks (Muhammed et al., 2024).

Machine learning (ML) has done well in crop recommendation and other agricultural decision-making supports, which means a big growth in that area is expected (Alam et al., 2025; Prity et al., 2024). Several articles that were done recently show that farming with the help of a ML-based soil and crop recommendation system that figures out the best crop for the location, soil, and weather of the farmer will reduce the risk of crop failure, and in general, improve farm management (Alam et al., 2025; Prity et al., 2024; Mahale et al., 2024). On the other hand, explainable recommendation researches point out that not only should valid agricultural systems be correct but also, they should be clear and actual enough to be used for real decision-making. Nevertheless, a recent article also points out that many of the crop recommendation systems still generate definite results while agricultural planning is highly influenced by uncertainties in the water availability, climate, and the concept of seasons (Mahale et al., 2024).

One other problem is that there is a big difference between using predictive models and doing optimization-based crop planning (Randall et al., 2024, 2022). Multi-objective optimization really has been used in agriculture for solving complex problems like crop rotation, cropping-structure design, land allocation, and water-efficient production, where different and even conflicting objectives had to be balanced rather than converted into a single numeric value. These research works demonstrate that optimization becomes vital during resource scarcity and changes in the climate however these frameworks in most cases are developed as independent mathematical models and not always combined with simple, scenario-based decision-support systems (Krityakierne et al., 2025).

Recent studies on agricultural decision support continue to emphasize on sustainable crop planning, which should integrate climatic variability, irrigation constraints, resource efficiency, and adaptive optimization among other factors, rather than only depend on static crop prediction models (Lioutas et al., 2026; Swati et al., 2026; Murad et al., 2026). The newly developed intelligent agricultural structures reveal that surrogate-assisted and scenario-aware optimization techniques are gaining more prominence in reducing computational complexity and supporting dynamic agricultural planning, even in uncertain environmental conditions (Elhoseny et al., 2026; Davcev et al., 2026). However, numerous systems mainly target only the improvement of prediction accuracy or consider optimization and recommendation as separate steps, which limits their suitability for practical district-level agricultural planning in changing climatic and resource-constrained conditions.

To fill this gap, this study selects CatBoost as the surrogate model in the proposed framework. Here, CatBoost is not used as the ordinary crop classifier; rather, it is used to imitate an expert-defined suitability function such that the crop evaluation can be performed efficiently under various agricultural scenarios. This surrogate modeling strategy based on CatBoost serves as a liaison between ML and multi-objective decision support, thereby allowing repeated crop evaluation without the need to recalculate the entire expert scoring logic for each scenario. This approach is very much in line with modern smart farming research, which is increasingly integrating AI, scenario-based planning, and resource-efficient optimization for decision-making in practice (Shams et al., 2024; Ikram et al., 2025; Ngimbwa et al., 2025).

Inspired by the shortcomings mentioned, this research has designed a CatBoost-powered surrogate-assisted, scenario-adaptive, multi-objective system for district-level crop selection based on agrometeorological, soil, and water-related parameters. The study does not view crop selection as a mere classification problem, but rather as a real planning issue where the crop suitability has to be balanced with water requirement, crop duration and the feasibility of crops under balanced, drought and short-season conditions, respectively. To begin with, a suitability score is empirically designed to represent agronomic preferences and operational constraints. Afterwards, CatBoost is used as the surrogate model to mimic that suitability behavior in a very efficient way. Last, a constraint-aware multi-objective ranking layer is used to find more practical crop alternatives for resource-sensitive planning. This representation is more aligned with the decision-support aspect of smart agricultural technology than a single-label crop prediction approach (Shams et al., 2024).

The main idea of this work is that the best crop based on suitability might not always be the best suggestion as the most useful one. It is possible that a crop with a very high suitability score in theory might be unattractive if it consumes too much water, has to be grown for a longer time, or gives bad results in limited scenarios. On the other hand, a recommendation that is more balanced may be willing to give up a little bit of raw suitability in order to provide better feasibility, water efficiency, and robustness. This research, through the combination of CatBoost-based surrogate modeling, feasibility-aware filtering, scenario adaptation, and multi-objective ranking, targets at delivering a crop-selection framework that is realistic and meaningful in the operation of district-wise agricultural planning (Aderele et al., 2025). Therefore, this study intends to fill the research gaps and develop a scenario-adaptive, surrogate training-based, and resource-aware agricultural decision-support system that can help district-level crop planning in changing environmental conditions. The system combines CatBoost surrogate training, an Improved ECA-DE solver, multi-criteria suitability modeling, and environmentally aware ranking to increase efficiency, human readability, and practical agricultural feasibility.

Based on the above discussion, the following research questions are formulated to guide the present study:

1. How can the district-wise crop recommendation methodology be transformed into a scenario-aware, multi-criteria decision-support problem rather than a single output prediction task?
2. Could a surrogate model based on CatBoost effectively imitate the expert-scored crop suitability for each of the agro-environmental scenarios to be used efficiently in crop recommendation?
3. Will a combined constraint-aware plus multi-criteria optimization-based framework outperform the resource efficiency and balance aspects of direct ranking and ML-only baseline methods?
4. Which crops perform well under balanced, drought, and short-season conditions, and how can their stability be helpful in reliable district-level agricultural planning?

Aligned with these research inquiries, this study makes the following contributions:

1. Instead of simple district-wise crop suggestions, this study used environmental awareness to perform multi-criteria decision analysis by considering agro-environmental factors such as suitability, water requirement, crop duration, and robustness.
2. Using CatBoost, this study constitutes surrogate modeling to replicate expert-defined suitability scores, thereby enabling efficient and scalable crop recommendations under diverse scenarios.
3. This study proposes a constraint-aware and scenario-adaptive framework that not only checks agronomic feasibility but also outputs balanced and practical crop selections through the optimization of criterion weights by proposed Improved ECA-DE.
4. It devises an extensive evaluation framework that includes a comparative analysis with baseline methods, scenario-based assessment, and a case study for exploring crop stability and district-level planning.

## 2 Related work

The latest studies in agricultural decision support demonstrate a significant shift toward data-driven crop recommendation systems that can assist farmers in selecting appropriate crops by utilizing soil, weather, and environmental data (Rani et al., 2023; Dey et al., 2024). Literature on Agriculture 4.0 and smart agriculture decision-support systems highlight that advanced agricultural advisory tool mainly depend on ML, sensor-generated data, and digital platforms to enhance crop planning and resource use (Kumar et al., 2024; Maffezzoli et al., 2022; Morales and Villalobos, 2023; Xu et al., 2021; Nyengere et al., 2026). Recent studies in 2026 show that present-day agricultural systems are tremendously moving in the direction of agricultural twin-transition set-ups that combine AI-powered data analysis with ecological digitalization plans for eco-friendly farming methods. Moreover, the latest smart farming systems mainly depend on 6G/IoT-enabled sensing networks and agentic AI-based agricultural advisory tools to help with live environment monitoring, adaptive crop planning, and autonomous agricultural decision-making (Lioutas et al., 2026; Elhoseny et al., 2026; Swati et al., 2026; Davcev et al., 2026).:Particularly in crop recommendation, fresh research has employed ML algorithms to correlate soil and climate factors with suitable crop options, and at the same time, highlight the potential of these systems in minimizing risk in crop selection (Alam et al., 2025; Prity et al., 2024; Rani et al., 2023).

Simultaneously, research also points out a major weakness of many crop recommendation systems, the majority of them function as single-output prediction models and completely overlook the aspects of feasibility, uncertainty, and operational trade-offs (Alam et al., 2025; Randall et al., 2022; Shams et al., 2024). Research on interpretable crop recommendation revealed that making agricultural AI understandable is becoming a matter of trust when farm-related decisions are influenced by recommendations. Increasing in on the quality of recommendations, smart agriculture research has demonstrated that besides accuracy, interpretability and actual usability in varying agricultural conditions are also very important criteria (Shams et al., 2024). Despite these rapid advancements, existing research models still lack interpretable multi-criteria surrogate optimization systems that can adapt dynamically to real-time climatic variability and district-level agricultural resource constraints without imposing excessive computational overhead. Most agricultural AI systems focus on prediction accuracy or optimization independently, and little attention has been paid to combining adaptive surrogate learning, scenario-aware optimization, and resource-constrained crop planning in a single intelligent agricultural decision-support setup.

Another line of research has explored multi-objective optimization for agricultural planning. These studies consider agricultural decision-making as a problem of balancing trade-offs rather than merely predicting outcomes. Recently, multi-objective optimization has been used for designing cropping structures, allocating agricultural lands, planning the water-energy-food nexus, and promoting sustainable agriculture (Agrawal et al., 2024; Sharma et al., 2025). The findings indicate that agricultural planning is a complex activity that needs to address multiple and often conflicting objectives such as maximizing production, using water efficiently, preserving the environment, choosing appropriate land, and ensuring that operations are feasible. This point is made even stronger by climate changes, when resource-aware planning becomes more beneficial than sticking to a single top-ranked crop (Gao et al., 2025). Recent climate-resilient agricultural frameworks emphasize the importance of intelligent resource adaptation, sustainability-aware optimization, and adaptive environmental decision-making under uncertain climatic conditions (Lioutas et al., 2026; Swati et al., 2026).

Another relevant point of reference is surrogate-assisted optimization, which is where a ML model helps to simulate a very costly or complex objective function so that the whole process can be run more efficiently (He et al., 2023; Liu et al., 2024). Surrogate-based multi-objective optimization has already been employed in agricultural landscape management and environmental planning problems in general, demonstrating that surrogate models are capable of significantly cutting down computational time while maintaining decision quality (Cruz et al., 2024). The most recent work has further advocated surrogate modeling as a viable way for constrained and robust multi-objective optimization. This is very much in line with the smart agricultural decision support scenario wherein the multi-scenario evaluations would typically turn out to be quite computationally expensive if done without the surrogate (Nguyen et al., 2019). Furthermore, recent intelligent agricultural systems increasingly rely on computationally efficient surrogate-assisted frameworks and autonomous AI-driven optimization mechanisms to support adaptive agricultural planning under dynamic climatic and operational uncertainty (Swati et al., 2026; Murad et al., 2026).

In summary, these research works have demonstrated that the academic community has made significant strides through three different strands of research: ML-based crop recommendation, multi-objective optimization for agricultural planning, and surrogate-assisted decision support. However, the integration of all three aspects within a single district-wise crop selection framework is still a rare focus of research. For example, very few articles have, on one hand, used a ML model like CatBoost, and on the other hand, have taken into account scenario-adaptive, constraint-aware, and multi-objective crop selection factors such as water and crop-duration in their study. In addition, existing agricultural AI solutions are devoid of interpretable and computationally efficient surrogate-assisted optimization setups that can dynamically adapt to climatic changes and resource constraints at the district level in real time. This deficiency is what inspired the current research. We argue that crop selection should not be treated merely as a classification problem but rather as a decision-support problem that reflects the changing agricultural scenarios.

### 2.1 Research gap

While prior research has played a significant role in crop recommendation, agricultural optimization, and surrogate-assisted decision support, some major research gaps remain to be addressed. For example, most of the systems that are currently in use tend to give greater emphasis to prediction accuracies and as such lag behind when it comes to planning crops adaptive to different scenarios in the face of changing weather conditions and availability of resources at the district level. In addition, only a handful of studies have been dedicated to the integration of interpretable surrogate learning, computationally efficient optimization, and multi-criteria decision-making in one agricultural decision-support setup. The drawbacks of the existing research prompted the development of our proposed system, which integrates CatBoost-based surrogate learning with Improved ECA-DE optimization for resource-efficient, district-specific, and climate-resilient crop recommendations.

## 3 Materials and methods

### 3.1 Study area and dataset description

**Figure 1:**
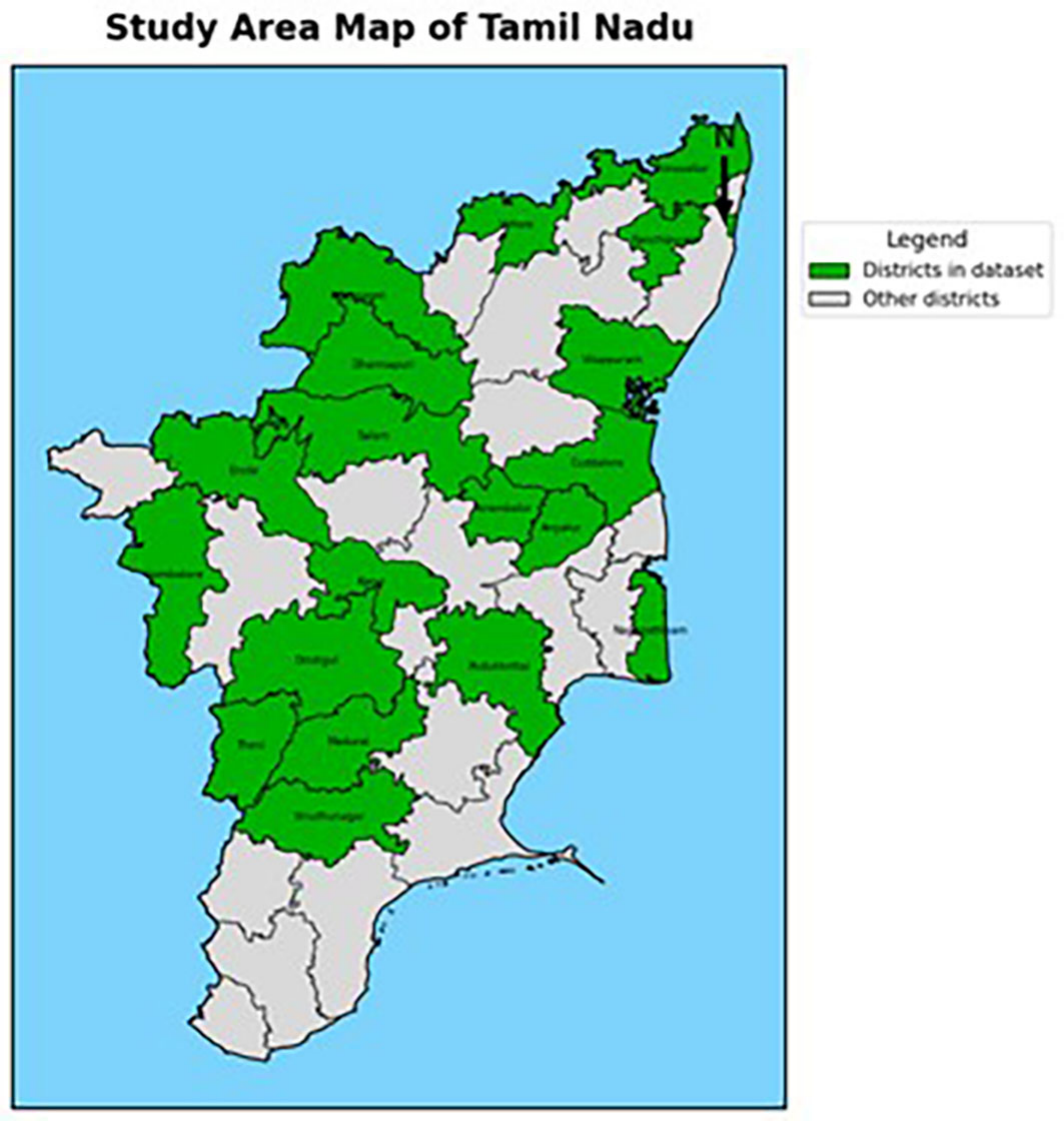
Study area for crop recommendation showing selected districts of Tamil Nadu.

The research was conducted in Tamil Nadu, one of the major agricultural states in southern India. Notably, Tamil Nadu has various soil properties, cropping environments, and meteorological conditions in different districts. The level of spatial variability in Tamil Nadu is a significant factor that can justify the idea of district-wise crop selection and smarter agricultural decision support. The area under consideration in this study is shown in **Figure 1**, which shows the districts that have been analyzed. The dataset used in this study was obtained from Mendeley Data (Seethapathy and Parvathi, 2020). The dataset description states that the data relate to the agro-meteorological factors affecting the growth of the main crops in Tamil Nadu and also contains information on the crops of the 37 districts of the state.

This dataset is publicly accessible in the Mendeley Data repository and was initially created for agro-environmental crop suitability analysis in Tamil Nadu. It merges district-level crop, soil, climatic, and water resource data derived from agricultural and environmental records. Because the data are arranged by district and not by individual farm, they are well suited for scenario-based agricultural planning, optimization, and decision-support applications. For this study, the final dataset includes 3437 instances and 20 features, such as district, crop name, crop type, soil properties, soil pH range, crop length, temperature range, water source, water requirement, and relative humidity.

Before starting the model development, preprocessing tasks such as checking for missing values, standardizing categories, verifying duplicates, and validating feature consistency were performed to enhance data reliability and make it more suitable for optimization and surrogate-model learning. Because the dataset provides information on crop-environmental suitability and resource requirements at the district level instead of repeated farm-level observations, it should be better understood as a district-wise crop suitability knowledge base. Such datasets are highly suitable for decision-making, ranking, and crop selection based on optimization methods. In addition, the use of a publicly accessible dataset improves the transparency, reproducibility, and practical applicability of the proposed agricultural recommendation framework.

### 3.2 Proposed work

**Figure 2:**
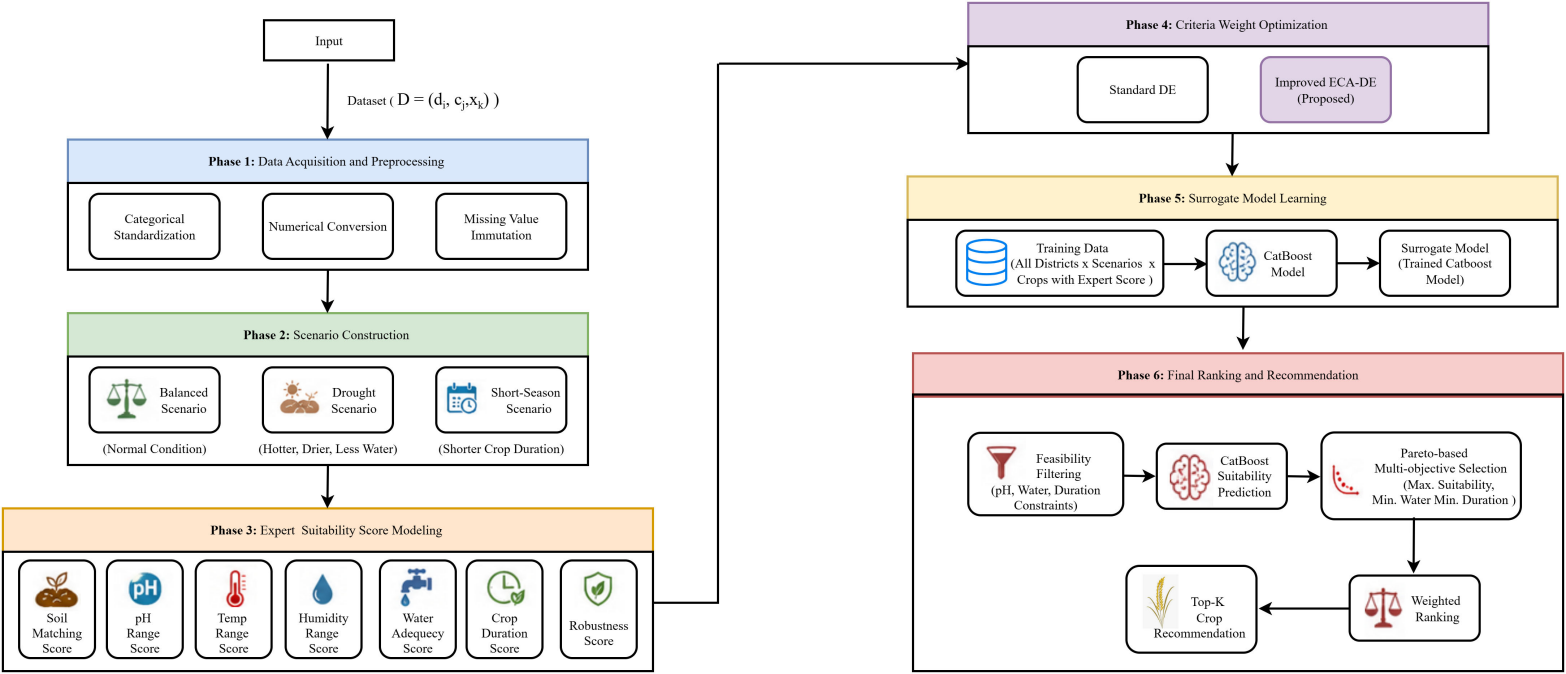
Architecture diagram of the proposed crop recommendation model.

The proposed work aims to develop a district-aware crop recommendation framework that integrates agronomic suitability modeling, scenario-based analysis, evolutionary optimization, surrogate learning, and constrained multi-criteria ranking. The biggest driver is that crop suitability varies not only across districts but also depends on local soil type, water source pH temperature, humidity, and crop duration conditions. Therefore, the framework builds district-specific scenarios, derives expert suitability scores, applies one Standard DE and one Improved ECA-DE to optimize the role of evaluation criteria, trains a surrogate suitability model with CatBoost, and recommends crops through a constrained ranking strategy. Considering that criterion-weight estimation is a nonlinear continuous optimization problem, the adoption of DE is quite backed up, and similarly, because the dataset includes mixed numerical and categorical agricultural attributes, the use of CatBoost is also natural. The overall architecture diagram is shown in **Figure 2** and its algorithm is shown in Algorithm 1. The steps involving the proposed work as follows;

Algorithm 1

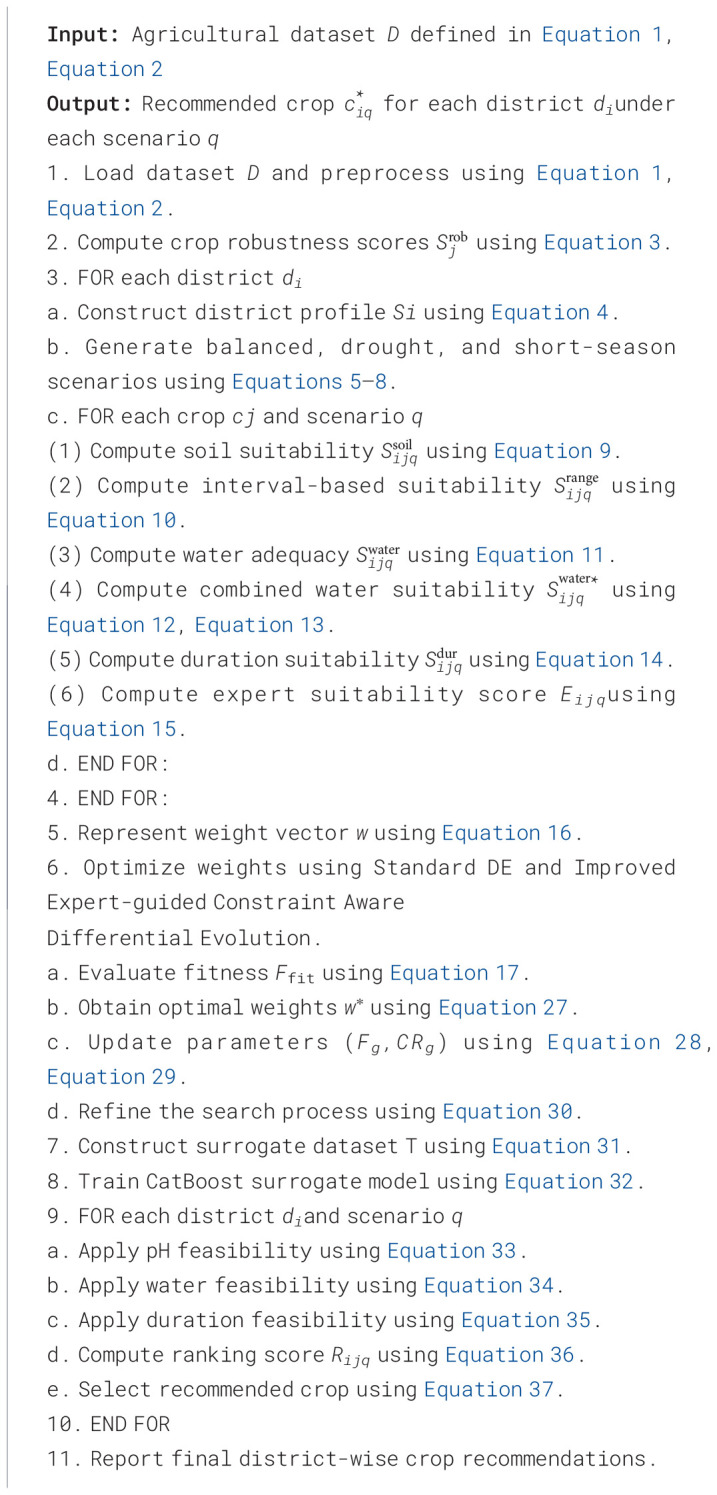

1. Data acquisition and pre-processing.
2. District-specific scenario construction.
3. Expert suitability score modeling.
4. Criteria-weight optimization using Standard DE and Improved ECA-DE.
5. CatBoost surrogate learning.
6. Constraint-based final ranking and recommendation.

#### 3.2.1 Phase 1: data acquisition and pre-processing

In the first phase, the agricultural dataset is illustrated as a district-crop representation:

(1) $$D=\{(d_{i},c_{j},X_{ij})\}$$

where $d_{i}$ represents the $i^{\text{th}}$ district, $c_{j}$ represents the $j^{\text{th}}$ crop, and $X_{ij}$ represents the corresponding agronomic input feature vector. The feature vector is represented as;

(2) $$X_{ij}=[x_{ij1},x_{ij2},\ldots ,x_{ijK}]$$

where K is the total number of agronomic features such as soil type, pH, temperature, humidity, water requirement, water source, and crop duration.

All categorical features are standardized, numerical variables were transformed to valid numeric forms, and incomplete records were deleted wherever needed. In addition, a crop robustness score ( $S_{j}^{rob}$) was calculated to measure stress tolerance over a wider range of environmental changes:

(3) $$S_{j}^{\text{rob}}=\frac{R_{j}^{\text{ph}}+R_{j}^{\text{temp}}+R_{j}^{\text{hum}}}{3}$$

where $R_{j}^{\text{ph}},\;R_{j}^{\text{temp}},\;R_{j}^{\text{hum}}$ indicates normalized adaptability widths for pH, temperature, and humidity, respectively.

During this phase, the dataset reliability for scenario generation and suitability modeling was confirmed. The inclusion of a robustness score can be justified by the fact that crops with a broader environmental tolerance are more likely to be viable even with variation at the district level.

#### 3.2.2 Phase 2: district-specific scenario construction

To reflect local agricultural heterogeneity, a district profile is constructed for each district:

(4) $$S_{i}=[s_{i1},s_{i2},s_{i3},s_{i4},s_{i5},s_{i6},s_{i7}]$$

where *s_i_*_1_ to *s_i_*_7_ represents dominant soil type, dominant water source, median soil pH, median temperature, median humidity, median water requirement, and median crop duration, respectively.

The three district-specific scenarios such as balanced scenario, drought scenario, short-season scenario was generated using district profile. The rules used for district-specific scenario generations are designed to represent realistic agricultural conditions commonly observed in crop planning and climate variability studies. These rules are based on agronomic reasoning rather than arbitrary assumptions.

The balanced scenario is defined as

(5) $$W_{i}^{\text{bal}}=\alpha_{b}\;s_{i6}$$

where *α_b_* is a water coefficient representing a balanced scenario. In this study, the value of *α_b_* is set to 1.20, which reflects a 20% increase in water availability over the district median value. This was meant to replicate a scenario of good agricultural conditions, with adequate irrigation, stable rainfall, and minimum water stress for crops. Because there is no climatic or seasonal pressure for early harvesting or shorter crop cycles, the preferred crop duration remains the same. Similarly, other environmental factors such as soil type, soil pH, temperature, and relative humidity were fixed at their district median values, which depict the typical conditions of the environment commonly found in the district. Therefore, the balanced scenario was the baseline cultivation condition against which the drought and short-season scenarios were compared for crop suitability analysis.

The drought scenario is defined in Equations 6 and 7;

(6) $$T_{i}^{\text{dr}}=s_{i4}+{\text{Δ}}_{T},\;H_{i}^{\text{dr}}=\text{max }(s_{i5}-{\text{Δ}}_{H},\;H_{\text{min}})$$

(7) $$W_{i}^{\text{dr}}=\alpha_{d}\;s_{i6},\;D_{i}^{\text{dr}}=\beta_{d}\;s_{i7}$$

where Δ*_T_* stands for temperature increment, Δ*_H_* denotes humidity reduction, *H*_min_ is the minimal humidity level, *α_d_* represents the drought water coefficient, and *β_d_* denotes the drought duration coefficient. Here, heat stress is simulated by a temperature increase of 2°C (Δ*_T_* = 2), and moisture content is reduced by 8% (Δ*_H_* = 8) with the minimum limit set to 20% (*H*_min_ = 20) to avoid unrealistic environmental conditions. Water availability is reduced to 70% of the district median value (*α_d_* = 0.70) to represent irrigation limitations and rainfall shortage, and the preferred crop duration is reduced to 90% of the normal duration (*β_d_* = 0.90) to support the selection of shorter-duration, drought-tolerant crops. If the original water source is not rainfed, the preferred water source in the scenario is adjusted toward rainfed conditions. Overall, these parameter settings simulate water stress and encourage the selection of drought-resistant crops.

The drought-related thresholds used in this study were derived from agro-meteorological investigations of climate variability and drought vulnerability in Tamil Nadu’s semi-arid agricultural regions (Varadan and Kumar, 2015), as well as climate risk assessments based on the work of the IPCC that cover a broader geographical area (IPCC, 2023). Backed by evidence, moderate temperature increases, drops in humidity, and restricted availability of water for irrigation are the main indicators of crop water stress, excessive evapotranspiration, and lack of agricultural productivity in drought-affected areas (Krishnan et al., 2026). For this reason, the temperature rises of $2^{\circ }$C, humidity drops of 8%, and 70% level of water availability that we have used as conditions represent, in a general sense, the climate stress situations that can be expected in Tamil Nadu for agricultural planning. They are not aimed at perfect duplication of a particular past drought in the region. The purpose of these scenario definitions was to create environmental stress conditions that were realistic to be a basis for the testing of the crop recommendation system that is scenario-aware and has been developed here, regarding its capability and performance under different conditions.

The short-season scenario $\left(W_{i}^{\text{ss}}\right)$ and the preferred duration $\left(D_{i}^{\text{ss}}\right)$ are defined as follows:

(8) $$W_{i}^{\text{ss}}=s_{i6},\;D_{i}^{\text{ss}}=\alpha_{s}\;s_{i7}$$

where *α_s_* controls the duration under short-season conditions. In this study, water availability is kept unchanged ( $W_{i}^{\text{ss}}=s_{i6}$) because short-season conditions are associated with limited cultivation time rather than actual water shortage. To support short-duration crops that can be harvested within the restricted growing period, the preferred crop duration is reduced to 75% of the district median duration (*α_s_*= 0.75). Soil type, soil pH, temperature, humidity, and the preferred water source are kept unchanged as district baseline values. This scenario enables the identification of crops that are better suited for regions with shorter planting periods, delayed monsoons, or constrained seasonal farming windows.

Short-season scenario parameters were derived from agronomic adaptation practices that were engaged in figuring out the best way to cope with the delayed monsoon onset, shortened cultivation windows, and seasonal cropping constraints as typical features of agricultural systems in Tamil Nadu under the influence of climate variability (Varadan and Kumar, 2015; Selvi et al., 2025) plus the assessment of climate change by the IPCC (on Climate Change, IPCC). The factor shortened crop-duration was unified primarily to prioritize crops capable of effecting their growth cycle within restricted seasonal windows while maintaining efficient resource utilization under climatic changing conditions.

#### 3.2.3 Phase 3: expert suitability score modeling

The criterion-wise suitability scores were computed for each *d_i_*, *c_j_*, and scenario *q* as follows. The soil matching score $\left(S_{ijq}^{\text{soil}}\right)$ is defined as:

(9) $$S_{ijq}^{\text{soil}}=\{\begin{array}{ll} 1, & \text{if exact soil match occurs}\\ 0.5, & \text{if partial soil match occurs}\\ 0, & \text{otherwise} \end{array}$$

For interval-based criteria such as pH, temperature, and humidity, The scores, $S_{ijq}^{\text{temp}}$, and $S_{ijq}^{\text{hum}}$ are computed using a range-membership function:

$S_{ijq}^{\text{ph}},\;S_{ijq}^{\text{temp}},\;S_{ijq}^{\text{hum}}\leftarrow S_{ijqk}^{\text{range}}$

(10) $$S_{ijqk}^{\text{range}}=\{\begin{array}{ll} 1, & \text{if }v_{iqk}\in [L_{jk},U_{jk}]\\ 1-\text{min }(\frac{\left|v_{iqk}-L_{jk}\right|}{U_{jk}-L_{jk}},\;\frac{\left|v_{iqk}-U_{jk}\right|}{U_{jk}-L_{jk}}), & \text{otherwise} \end{array}$$

where $v_{iqk}$ is the scenario value of district *d_i_*for criterion *k*, and *L_jk_*, *U_jk_*are the lower and upper acceptable bounds of crop *c_j_*.

The water adequacy score $\left(S_{ijq}^{\text{water}}\right)$ is computed as:

(11) $$S_{ijq}^{\text{water}}=\{\begin{array}{ll} 1, & \text{if }R_{j}^{\text{water}}\leq A_{iq}^{\text{water}}\\ 1-\frac{R_{j}^{\text{water}}-A_{iq}^{\text{water}}}{R_{j}^{\text{water}}}, & \text{if }R_{j}^{\text{water}}>A_{iq}^{\text{water}} \end{array}$$

where $R_{j}^{\text{water}}$ is the average water requirement of crop *c_j_*, and $A_{iq}^{\text{water}}$ is the available water for district *d_i_*under scenario *q*.

The combined water-related score $\left(S_{ijq}^{\text{water}*}\right)$, which integrates water adequacy and source compatibility, is defined as:

(12) $$S_{ijq}^{\text{water}*}=\lambda_{1}S_{ijq}^{\text{water}}+\lambda_{2}S_{ijq}^{\text{wsrc}}$$

where *λ*_1_ and *λ*_2_ assign greater importance to water sufficiency compared to source matching. The water-source matching score $\left(S_{ijq}^{\text{wsrc}}\right)$ is given by:

(13) $$S_{ijq}^{\text{wsrc}}=\{\begin{array}{ll} 1, & \text{if exact source match occurs}\\ 0.5, & \text{if partial source match occurs}\\ 0, & \text{otherwise} \end{array}$$

The duration suitability score $\left(S_{ijq}^{\text{dur}}\right)$ is calculated as:

(14) $$S_{ijq}^{\text{dur}}=1-\frac{\left|D_{j}-D_{iq}\right|}{max\;(|D_{iq}|,1)}$$

where *D_j_*is the average crop duration and *D_iq_*is the scenario-preferred duration.

The final expert suitability score (*E_ijq_*) is obtained using weighted aggregation:

(15) $$E_{ijq}=w_{1}S_{ijq}^{\text{soil}}+w_{2}S_{ijq}^{\text{ph}}+w_{3}S_{ijq}^{\text{temp}}+w_{4}S_{ijq}^{\text{hum}}+w_{5}S_{ijq}^{\text{water}*}+w_{6}S_{ijq}^{\text{dur}}+w_{7}S_{ijq}^{\text{rob}}$$

subject to:


$$w_{k}\geq 0,\;\sum_{k=1}^{7}w_{k}=1$$

where *w*_1_*,w*_2_*,…,w*_7_ are the criterion weights.

This phase provides an interpretable agronomic scoring mechanism. The constants in the soil and water-related scoring functions act as expert preference levels rather than learned parameters.

#### 3.2.4 Phase 4: criteria-weight optimization using standard DE and improved ECA-DE

The expert suitability score in Equation 18 depends strongly on the weights *w*_1_*,w*_2_*,…,w*_7_. If these weights are assigned manually, the resulting recommendations may become subjective and inconsistent across district-specific scenarios. Therefore, this phase formulates criterion weighting as an optimization problem. A candidate weight vector *w* is represented as:

(16) $$w=[w_{1},w_{2},\ldots ,w_{7}]$$

The present work uses Standard DE as a baseline optimizer and proposes an Improved ECA-DE as the main research contribution.

##### 3.2.4.1 Standard DE

Standard DE is a population-based evolutionary optimization algorithm that solves continuous optimization problems with iterative operations of mutation, crossover, and selection. The idea of this algorithm is to maintain a population of candidate solutions and improve them by creating trial vectors using a scaled difference of randomly selected individuals (Storn and Price, 1997). Because of its robustness, simplicity, ease of implementation, and derivative-free search, the Standard DE has been broadly adopted in many engineering optimization problems, feature selection, neural network training, and parameter tuning applications. In this, the mutation operation forms a so-called mutant vector from the existing members of the population, and the crossover operation uses the mutant and target vectors to create new trial vectors. Subsequently, better choices are selected. The process that occurs here allows an efficient search in complex continuous spaces without the need to incorporate gradient information. In this study, the standard DE is used as a benchmark optimization method to identify the criterion weights for expert suitability scoring. The algorithm attempts to discover an optimal weight vector that yields the maximum suitability scoring performance for district – crop – scenario combinations. It was used as a standard baseline for examining the proposed Improved ECA-DE framework.

Although the Standard DE is a general optimizer, there are still some shortcomings in applying it to an agri-recommendation system. First, the Standard DE fitness formulation is quite general and does not explicitly integrate agricultural sustainability concerns, such as water conservation, crop duration minimization, robustness to uncertainty, or specific-agriculture scenario adaptation. Second, the mutation and crossover operations are fixed using constant parameters during the entire online optimization trajectory. Large parameter values may induce an unstable search behavior, whereas small values seem to lack the global search ability. Third, the Standard DE suffers from premature convergence when the population diversity declines rapidly in later generations. This may result in the search being trapped in local optima and the use of poor criterion weights. Fourth, the algorithm is not domain-guided and treats all search dimensions in the same way, regardless of the relative degree of importance of individual criteria to agriculture. Fifth, the Standard DE does not explicitly preserve intuitively balanced or interpretable weight distributions, which might cause the generation of overly stressed weights that hamper the transparency of recommendations and their usability. Another limitation of the standard DE is that it does not explicitly promote the stability of the solutions over the different agronomic scenarios such as balanced, drought, and short season. Therefore, the solutions optimized using the standard DE could differ significantly across scenarios, which could affect the recommendation stability. In addition, the standard DE has no feature of rewarding any secondary agronomic condition such as soil compatibility, fogging insensibility to stress, robustness, etc.

To overcome these problems, several domain-adaptive improvements were proposed in the proposed Improved ECA-DE: (1) an agronomy-aware multi-component fitness function that can effectively maximize the objectives of suitability discrimination, water use efficiency, crop duration efficiency, and recommendation stability, together with the domain-sensitive weight term; (2) adaptive mutation and crossover schedules are incorporated to dynamically balance exploration and exploitation during different optimization stages; (3) the guided search strategy biases the population toward the optimal solution, which enhances the quality of convergence and efficiency of the optimization; (4) a stagnation treatment and diversity-preservation strategy are devised to reinitialize the inferior solutions while the population improvement becomes stagnant, which enables the algorithm to escape from local optimization; (5) the dominance penalties and secondary offensive components contribute to the avoidance of overemphasis on only one criterion and to the preservation of the utility of the secondary agricultural factors. The proposed Improved ECA-DE turns weighted optimization into a domain-driven agricultural optimization process instead of a general mathematical optimization problem; therefore, the resulting crop recommendations will be less sensitive to instabilities, more interpretable, resource-efficient, and practically suitable for district-specific scenario-based agricultural planning.

##### 3.2.4.2 Proposed improved ECA-DE

**Figure 3:**
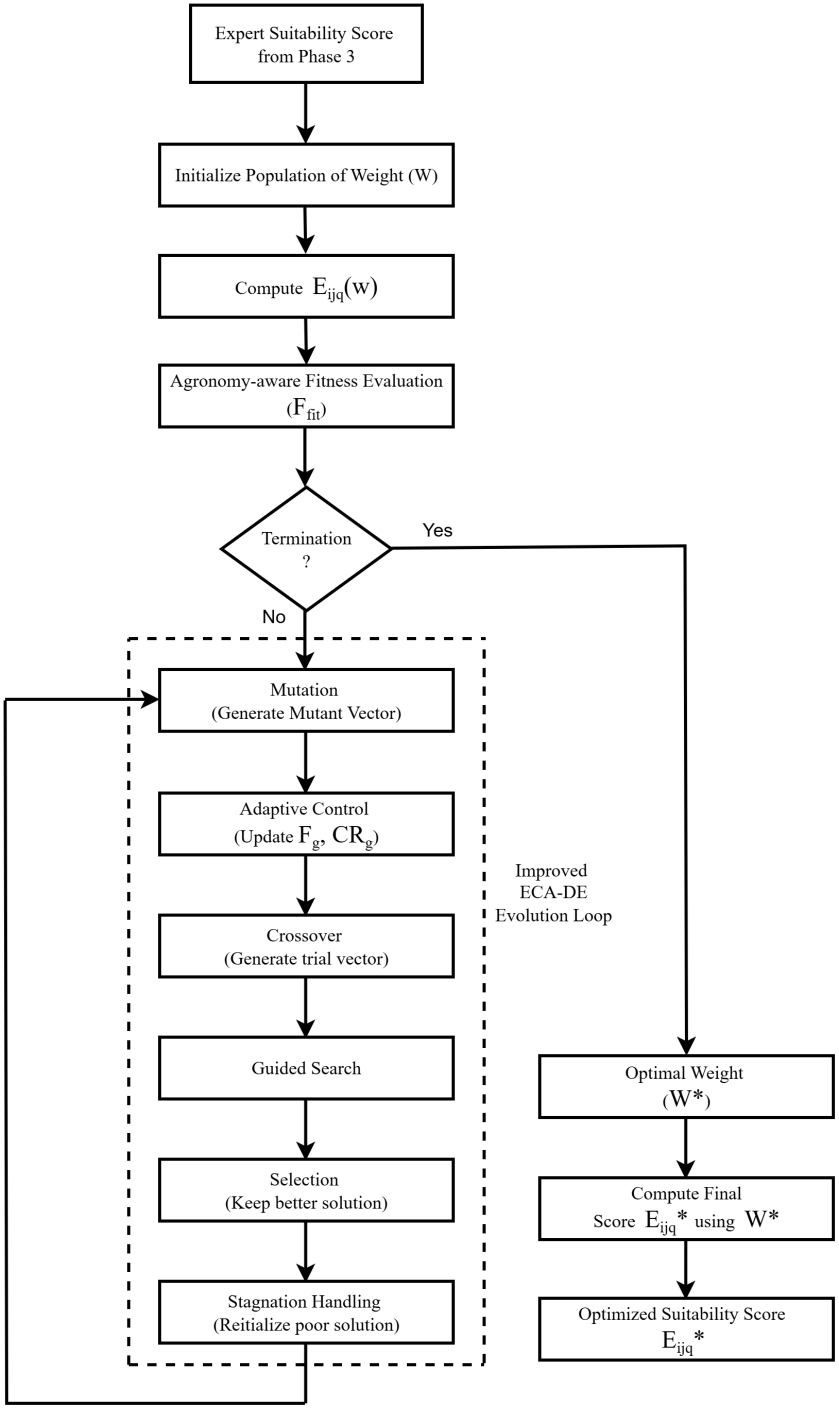
Flowchart of the proposed improved ECA-DE.

The proposed Improved ECA-DE extends Standard DE by incorporating three main enhancements are agronomy-aware fitness design, adaptive mutation, crossover control, and guided search, diversity preservation. The flowchart of the proposed improved EDA-DE is as shown in **Figure 3**.

The fitness (*F*_fit_) of a candidate weight vector is evaluated using;

(17) $$F_{\text{fit}}=\phi_{1}F_{\text{spread}}+\phi_{2}F_{\text{water}}+\phi_{3}F_{\text{dur}}+\phi_{4}F_{\text{stab}}+\phi_{5}F_{\text{bonus}}+\phi_{6}F_{\text{bal}}+\phi_{7}F_{\text{sec}}+\phi_{8}P_{\text{dom}}$$

The score-spread component $\left(F_{\text{spread}}\right)$ encourages discrimination between more and less suitable crops and is defined as:

(18) $$F_{\text{spread}}=\sigma (E_{ijq})$$

where *σ*(·) denotes the standard deviation of suitability scores across all district–crop–scenario combinations. A larger spread indicates stronger discrimination among candidate crops.

The water-efficiency component (*F*_water_) rewards water-efficient ranking behavior and is given in Equation 19:

(19) $$F_{\text{water}}=\text{corr}(1-{\hat{W}}_{j},\;E_{ijq})$$

where ${\hat{W}}_{j}$ is the normalized water requirement of crop *c_j_*, and corr(·) denotes the correlation operator.

The duration-efficiency component (*F*_dur_) rewards shorter-duration crops and is defined in Equation 20:

(20) $$F_{\text{dur}}=\text{corr}(1-{\hat{D}}_{j},\;E_{ijq})$$

where ${\hat{D}}_{j}$ denotes normalized crop duration.

The scenario stability component (*F*_stab_) promotes consistency across scenarios and is defined in Equation 21:

(21) $$F_{\text{stab}}=\frac{1}{1+\sigma ({\bar{E}}_{q})}$$

where ${\bar{E}}_{q}$ is the mean suitability score under scenario *q*, and $\sigma ({\bar{E}}_{q})$ is the standard deviation across scenarios.

The scenario bonus (*F*_bonus_) rewards scenario-adaptive recommendations and is given in Equation 22:

(22) $$F_{\text{bonus}}=\frac{1}{Q}\sum_{q=1}^{Q}F_{\text{bonus},q}$$

where *Q* is the number of scenarios. For each scenario *q*, let *L_q_*denote the average low-water preference and *S_q_*denote the average short-duration preference among top-ranked crops. Then the scenario wise bonus is given in Equation 23:

(23) $$F_{\text{bonus},q}=\{\begin{array}{ll} \rho_{1}L_{q}+\rho_{2}S_{q}, & \text{for drought scenario}\\ \rho_{3}L_{q}+\rho_{4}S_{q}, & \text{for other scenarios} \end{array}$$

The balance component $\left(F_{\text{bal}}\right)$ encourages evenly distributed weights and is given in Equation 24:

(24) $$F_{\text{bal}}=1-\sigma (w)$$

The secondary-support component $\left(F_{\text{sec}}\right)$ preserves contributions from secondary criteria and is given in Equation 25:

(25) $$F_{\text{sec}}=w_{\text{soil}}+w_{\text{ph}}+w_{\text{temp}}+w_{\text{hum}}+w_{\text{rob}}$$

The dominance penalty $\left(P_{\text{dom}}\right)$ penalizes excessive weight concentration and is given in Equation 26:

(26) $$P_{\text{dom}}=\text{max }(0,\text{ max }(w)-0.45)$$

The optimal weight vector is obtained as:

(27) $$w^{*}=\text{arg }\underset{w}{\max}\;F_{\text{fit}}(w)$$

The Improved ECA-DE uses adaptive parameter control for mutation factor and crossover rate:

(28) $$F_{g}=F_{\text{max}}-\frac{(F_{\text{max}}-F_{\text{min}})g}{G-1}$$

(29) $$CR_{g}=CR_{\text{min}}+\frac{(CR_{\text{max}}-CR_{\text{min}})g}{G-1}$$

These schedules encourage exploration in early generations and refinement in later stages.

To enhance exploitation, the search is biased toward the best solution:

(30) $$v_{p}^{'(g)}=0.7\;v_{p}^{\left(g\right)}+0.3\;w^{\text{best}}$$

where $v_{p}^{'(g)}$ is the refined mutant vector, $v_{p}^{\left(g\right)}$ is the original mutant vector, and $w^{\text{best}}$ is the best solution found so far.

Furthermore, a stagnation-handling scheme is implemented, which allows poor solutions to be reinitialized when progress stops. This, in turn, saves the diversity of the population while also addressing the problem of premature convergence. Overall, the proposed Improved ECA-DE is better aligned with this problem than the Standard DE, mainly because it employs an agronomy-aware fitness function instead of a purely generic objective. In addition, it adjusts mutation and crossover during the search, uses the current best solution as a guide for the search, involves diversity preservation when stagnation occurs, and most importantly, expresses water efficiency, duration shortening, stability, and interpretability directly. Therefore, Phase 4 is where the main contribution of this study lies, as it changes the criterion weighting from a subjective decision to a domain-adapted evolutionary optimization problem.

#### 3.2.5 Phase 5: CatBoost surrogate learning

Once the optimized expert suitability scores are obtained, they are used to generate surrogate training data. Let the training set be represented as

(31) $$T={\left\{(z_{n},y_{n})\right\}}_{n=1}^{P}$$

where *z_n_*is the input feature vector containing district, crop, and scenario information, *y_n_*is the expert suitability score from Equation 18, and *P* is the total number of training samples.

The CatBoost model learns the mapping:

(32) $${\hat{y}}_{n}=f_{\text{CB}}(z_{n})$$

where *f*_CB_ is the trained CatBoost regressor, and ${\hat{y}}_{n}$ is the predicted suitability score.

The created surrogate dataset was divided in the ratio of 80:20 for training and testing purposes, respectively. While training the CatBoost surrogate model was carried out with the training portion, the testing part was used for the prediction performance assessment. The reliability, consistency, and ability of the surrogate model to generalize under diverse data partitions were further measured using 5-fold cross-validation. To ensure that the experimental results could be reproduced and remained consistent, a constant random seed was used even during data splitting.

This step allows the expert scoring logic to be modelled by a ML system that can effectively deal with mixed categorical and numerical features. In addition, it makes the system more scalable because, after training, the substitute model can swiftly estimate the suitability for all district-scenario combinations.

#### 3.2.6 Phase 6: constraint-based final ranking and recommendation

In the final phase, crops are filtered by practical agronomic feasibility constraints before ranking. The pH feasibility condition is defined as

(33) $$PH_{j}^{\text{low}}-\delta_{\text{ph}}\leq PH_{iq}\leq PH_{j}^{\text{high}}+\delta_{\text{ph}}$$

where $PH_{j}^{\text{low}}$ and $PH_{j}^{\text{high}}$ are the lower and upper acceptable pH bounds of crop $c_{j}$, $PH_{iq}$ is the scenario pH of district $d_{i}$, and $\delta_{\text{ph}}$ is the pH tolerance margin.

The water feasibility condition is:

(34) $$R_{j}^{\text{water}}\leq A_{iq}^{\text{water}}$$

and the crop-duration feasibility condition is:

(35) $$D_{j}\leq D_{iq}+\delta_{D}$$

where *D_j_*is the duration of crop *c_j_*, *D_iq_*is the preferred duration of district *d_i_*under scenario *q*, and *δ_D_*is the allowable duration margin.

After feasibility filtering, the final ranking score (*R_ijq_*) is computed as:

(36) $$R_{ijq}=\omega_{1}{\tilde{S}}_{ijq}+\omega_{2}{\tilde{W}}_{ijq}+\omega_{3}{\tilde{D}}_{ijq}$$

where ${\tilde{S}}_{ijq}$ is the normalized predicted suitability, ${\tilde{W}}_{ijq}$ is the normalized inverse water requirement, ${\tilde{D}}_{ijq}$ is the normalized inverse crop duration, and *ω*_1_, *ω*_2_, *ω*_3_ are the ranking weights.

The final recommended crop is selected using Equation 37:

(37) $$c_{iq}^{*}=\text{arg }\underset{j}{\max}\;R_{ijq}$$

where $c_{iq}^{*}$ denotes the top-ranked crop for district *d_i_*under scenario *q*.

This phase guarantees that the suggestion is not only based on suitability prediction but also on practical agricultural problems, such as water needs and time for a crop to grow. In general, this study attempts to implement scenario generation specific to a certain district, interpretable expert suitability modeling, Improved ECA-DE based criterion weight optimization, CatBoost surrogate learning, and constraint-based final ranking into a single crop recommendation framework. The entire method is district-aware, transparent, and future-oriented. A significant advantage of this approach is that it combines agri-farming knowledge with adaptive optimization and ML. Therefore, the recommendations are not only suitable but also help save resources and are feasible in terms of operation.

## 4 Results and discussion

### 4.1 Experimental setup and data summary

The proposed framework was evaluated on an agricultural dataset specifying districts with features such as soil pH, temperature, humidity, water, and crop duration. Data after cleaning were used to create three different scenarios such as balanced, drought, and short-season for each district individually. The main data properties and scenarios are described in **Tables 1**, **2**, respectively. Each district’s data were assessed against various scenarios and crops; hence, there was a considerable increase in the final input set for surrogate training. This allowed the capture of expert suitability pattern development on an extensive scale. The introduction of district-level scenarios is important because it enables the suggestion system to consider local differences in the environment and farming practices rather than a uniform scenario for all districts. **Table 3** outlines the hyperparameter settings were selected to ensure stable surrogate model learning, balanced optimization performance, and fair comparative evaluation between the standard DE and the proposed ECA-DE. The same CatBoost configuration and optimization settings were consistently maintained across all agricultural scenarios to avoid experimental bias, and ensure reproducibility and methodological consistency.

**Table 1:** Experimental dataset and scenario summary.

Item	Value
Dataset shape	3437 × 20
Number of districts	37
Number of crops	105
Number of scenarios per district	3
Scenario types	Balanced, Drought, Short-season
Surrogate training data shape	381,507 × 38

**Table 2:** District-specific scenario settings (I: Balanced, II: Drought, III: Short-season).

District	Scenario	User soil	User pH	User temp	User humidity	Available water	Pref. duration	Pref. water source
Ariyalur	I	Sandy loam	6.5	24.00	80.0	1260.0	90.0	Both
Ariyalur	II	Sandy loam	6.5	26.00	72.0	735.0	81.0	Rainfed
Ariyalur	III	Sandy loam	6.5	24.00	80.0	1050.0	67.5	Both
Coimbatore	I	Sandy loam	6.5	24.75	80.0	1200.0	90.0	Both
Coimbatore	II	Sandy loam	6.5	26.75	72.0	700.0	81.0	Rainfed
Coimbatore	III	Sandy loam	6.5	24.75	80.0	1000.0	67.5	Both
Chengleput	I	Sandy loam	6.5	24.00	80.0	1266.0	90.0	Both
Chengleput	II	Sandy loam	6.5	26.00	72.0	735.0	81.0	Rainfed
Chengleput	III	Sandy loam	6.5	24.00	80.0	1050.0	67.5	Both

**Table 3:** Hyperparameter settings used in the proposed framework.

Hyperparameter	Setting/Range
CatBoost iterations	300
CatBoost tree depth	6
CatBoost learning rate	0.05
Standard DE generations	40
Standard DE population size	20
Mutation factor (F)	0.5 – 1.0
Recombination rate (CR)	0.7
Improved ECA-DE generations	40
Improved ECA-DE population size	20
Adaptive mutation factor (F)	0.9 → 0.4
Adaptive crossover rate (CR)	0.4 → 0.9

### 4.2 Distribution of input agronomic variables

**Figure 4:**
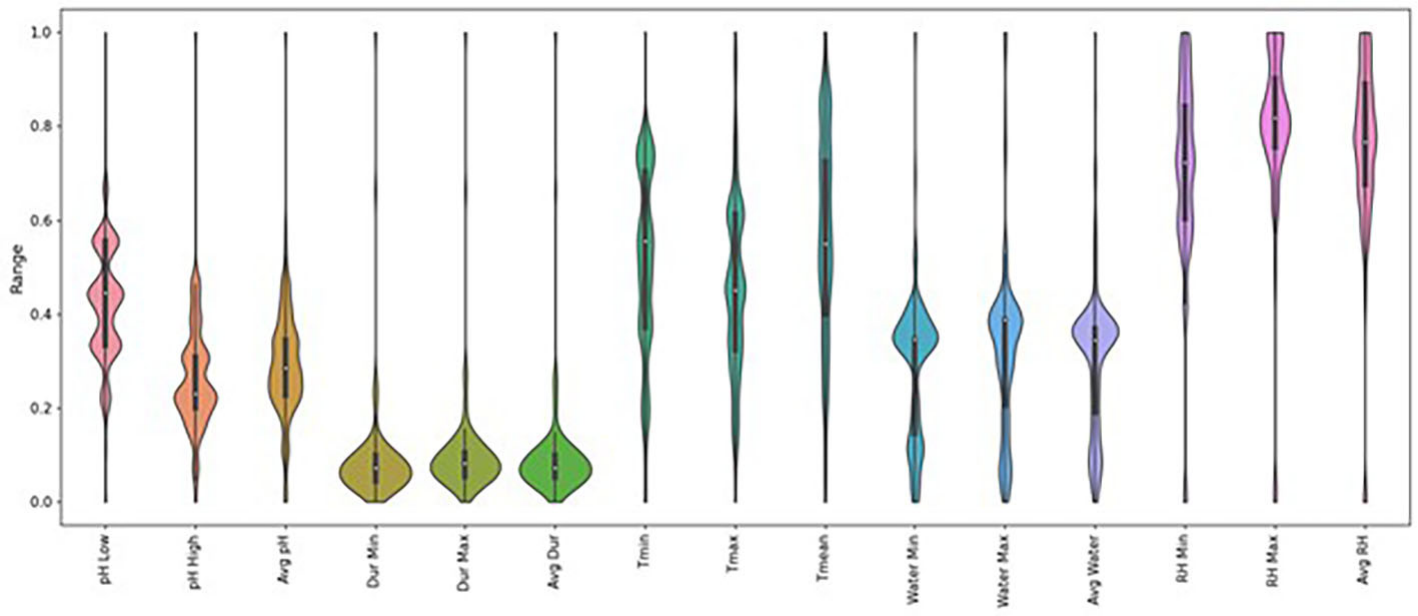
Input variable distribution.

The distributions of the standardized agronomic features considered in the framework are shown in **Figure 4**. According to the violin plot, pH-related variables were mainly found within relatively narrow ranges, whereas temperature, humidity, and water-related variables had wide distributions. Crop-duration variables are also, to some extent, grouped at lower and moderate values. This indicates that the dataset contains a good number of short- and medium-duration crops.

### 4.3 Performance comparison of standard DE and proposed improved ECA-DE

The proposed Improved ECA-DE, standard DE, and other adaptive DE variants such as JADE (Adaptive DE with Optional External Archive), SaDE (Self-adaptive DE) and SHADE (Success-History based Adaptive DE) were compared in the optimization phase to determine the best criterion weights for the expert suitability model. **Table 4** shows that Standard DE, JADE, SaDE, and SHADE obtained almost the same best fitness values, whereas the Improved ECA-DE Obtained a fitness value close to 0. 402195. Even if the fitness difference is negligible, the Improved ECA-DE managed to be better in terms of computational efficiency by reducing the execution time from 99,576.65 s to 10,836.29 s and memory consumption from 773.14 MB to 360.62 MB than Standard DE. JADE and SHADE also performed well in terms of execution time and memory usage, whereas SaDE required more execution time. Therefore, the suggested Improved ECA-DE results in a better combination of optimization accuracy, execution time, and memory efficiency, making it more appropriate for agricultural decision-support applications.

**Table 4:** Comparison of optimization performance between standard DE, improved ECA-DE and variants of DE.

Optimizer	Best fitness	Execution time (sec)	Memory (MB)
Standard DE	0.402245	99576.65	773.14
Improved ECA-DE	0.402195	10836.29	360.62
JADE	0.402245	11143.83	361.08
SaDE	0.402245	36060.19	360.66
SHADE	0.402244	11110.66	360.63

### 4.4 Surrogate model performance

**Figure 5:**
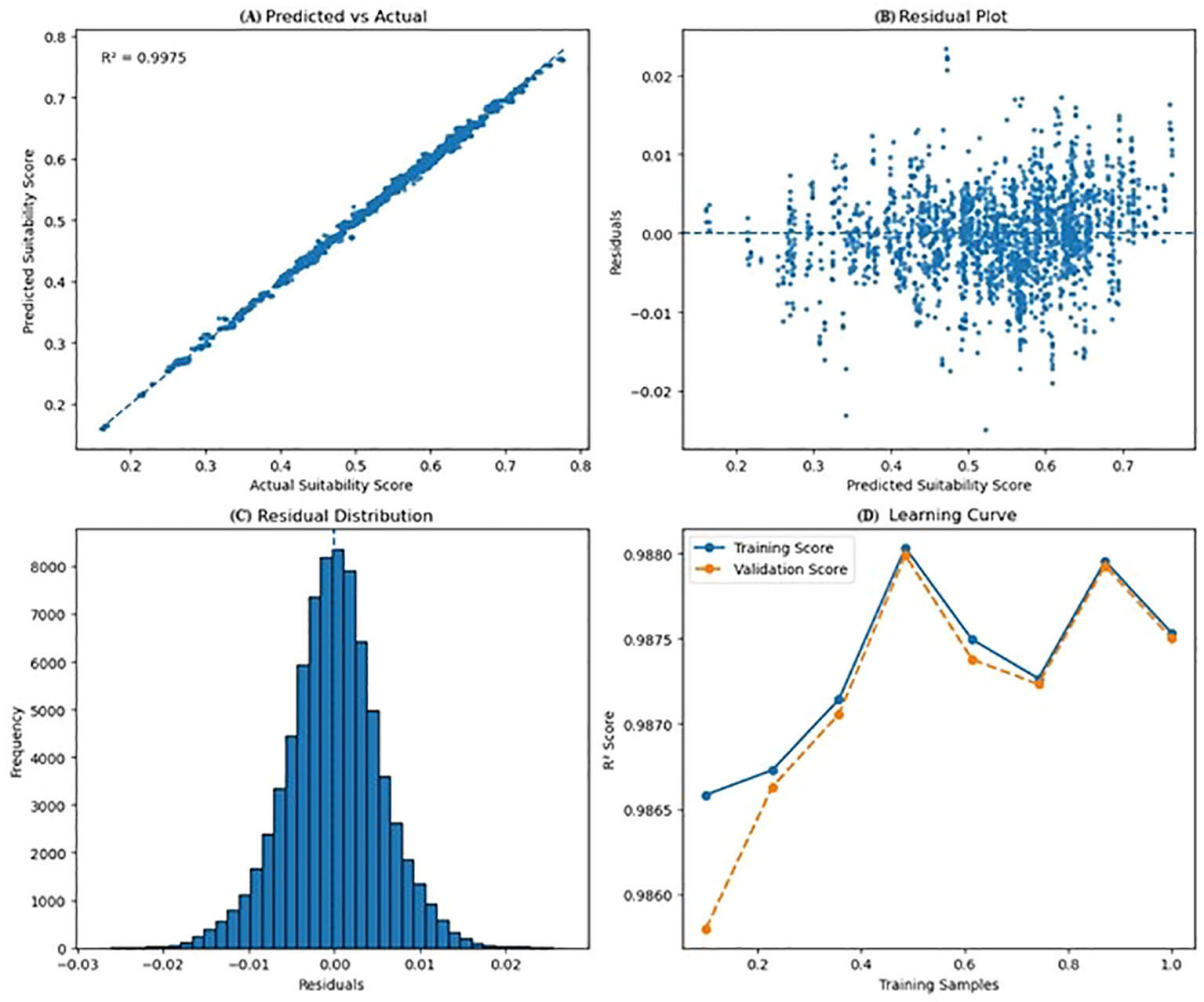
Comprehensive validation of the surrogate model. **(a)** Predicted vs Actual, **(b)** Residual Plot, **(c)** Residual Distribution, and **(d)** Learning Curve.

The performance of the surrogate model is illustrated in **Figure 5**, which includes a prediction versus observed plot, residual analysis, and learning curve. In the **Figure 5A**, the predictions aligned very well along the best-fit line, which illustrated a very good correlation with the actual values (*R*^2^ = 0.9975). The distribution of the residuals in **Figure 5B** showed no dependence on the predicted value and illustrated all residuals centered around zero without any structure. The distribution of the residuals has a symmetric shape centered around 0, as shown in **Figure 5C**. The **Figure 5D** could help analyze how well the model would fit the subject data, and studies in this figure could find that the training and validation performances had both high values and small gaps.

Before surrogate-model training, the generated dataset of 381,507 samples was separated into training and testing sets based on an 80:20 split ratio. Meanwhile, 5-fold cross validation was used to test the stability, robustness, and generalization of the CatBoost surrogate model on various data partitions. A constant random seed was applied to the dataset separation for the purpose of repeatability. In addition, the overfitting problem was alleviated by employing CatBoost’s built-in regularization and gradient boosting activities, as well as performing independent testing and learning-curve monitoring. As presented in **Figure 5D**, the training and validation scores hardly move away from one another at various training sizes with odds, in fact, very small performance gaps, indicating a very stable generalization behavior with practically no overfitting. The residual plots in **Figures 5B, C** show no strong repetitive patterns and are evenly spread around zero, which also supports the assertion of the high accuracy and dependability of the surrogate model.

It is important to note that the CatBoost model within the proposed architecture is a substitute learning model that is trained to mimic the deterministic expert-defined suitability scoring function rather than real-world noisy biological crop yield observations. Therefore, the reported *R*^2^ value mainly represents the computational fidelity and approximation capability of the surrogate model with the expert suitability function that has been formulated. The excellent predictive performance indicates that the surrogate model is capable of learning the optimization behavior of the expert-defined scoring mechanism and can replicate it very well, which in turn, helps multi-scenario agricultural suitability evaluation that is computationally efficient. These preliminary evaluation results confirmed that the surrogate was accurate, stable, not overfitted or underfitted, and significantly applicable to on-farm prediction.

### 4.5 Comparative analysis across methods

The practical effectiveness of the proposed district-aware crop recommendation framework was first compared with five other baseline methods: CatBoost Only, CatBoost + Constraints, Expert Weighted Ranking, Weighted Sum Ranking, and Pareto Only. The CatBoost-only method depends solely on prediction without considering real-world constraints, whereas CatBoost + Constraints adds key agronomic factors, such as soil pH, water availability, and crop duration. Expert Weighted Ranking uses the weights optimized with the Improved ECA-DE, whereas Weighted Sum Ranking uses a fixed mixture of criteria. The Pareto Only method is dedicated to the multi-objective trade-offs between suitability, water requirement, and crop duration, but it does not provide a final ranking. These methods represent various recommendation strategies, such as prediction-based, rule-based, optimization-based, and multi-objective approaches. The comparison was performed using the main indicators, such as average water requirement, crop duration, and suitability score over districts and scenarios, thus offering a thorough evaluation of both the model performance and practical applicability in agriculture.

**Figure 6:**
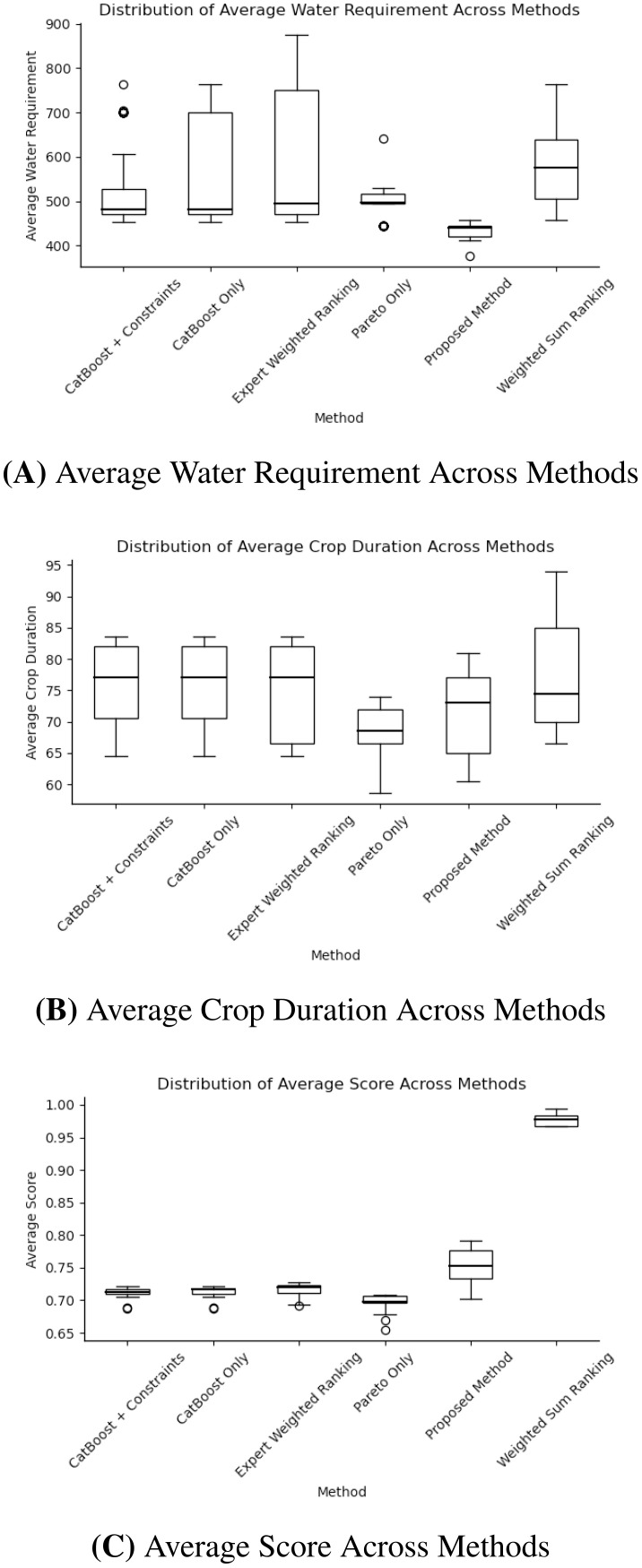
Comparative analysis of **(A)** average water requirement, **(B)** crop duration, and **(C)** suitability score across different methods.

A performance comparison of various recommendation methods in all districts is shown in **Figures 6A–C**. The figures focus on the significant trade-offs in water requirements, crop duration, and suitability score. **Figure 6A** shows that the proposed method not only requires the least amount of water but also maintains very constant water requirements (420 mm - 450 mm) as opposed to other methods that are less consistent or require more water. CatBoost Only and Expert Weighted Ranking were the two methods that were the most variable and demanded more water.

In terms of crop duration as shown in **Figure 6B**, the proposed method has the advantage of keeping the number of days moderate and very stable (65–80 days), unlike the Weighted Sum Ranking, which varies more and has longer durations. Moreover, in **Figure 6C** reveals that although the Weighted Sum Ranking results in the highest average suitability scores (0.97 - 0.99), it does so through a trade-off between using more water and being more variable. In contrast, the proposed method shows a moderate increase in the score (0.73 - 0.78) compared to the baseline methods such as CatBoost Only and Pareto Only (0.69 - 0.72). Overall, the findings highlight the proposed framework as a capable one that balances suitability, resource efficiency, and stability, and thus it is more viable and dependable for agricultural decision-making in real life.

### 4.6 Case study analysis: Ariyalur district under balanced scenario

**Figure 7:**
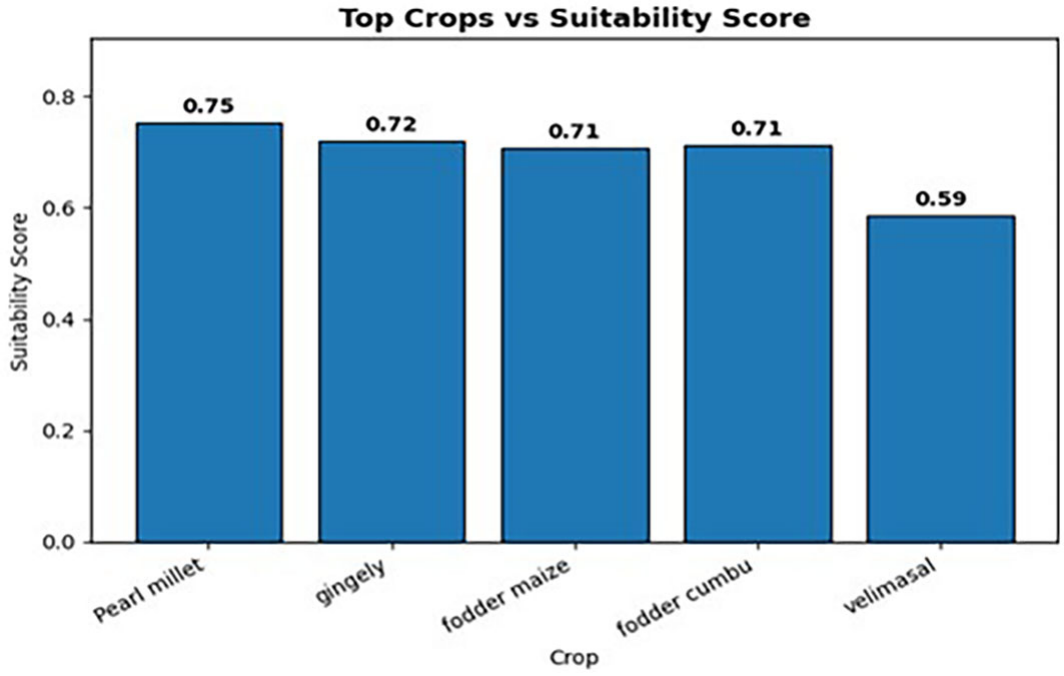
Top crop recommendations based on suitability scores.

**Figure 8:**
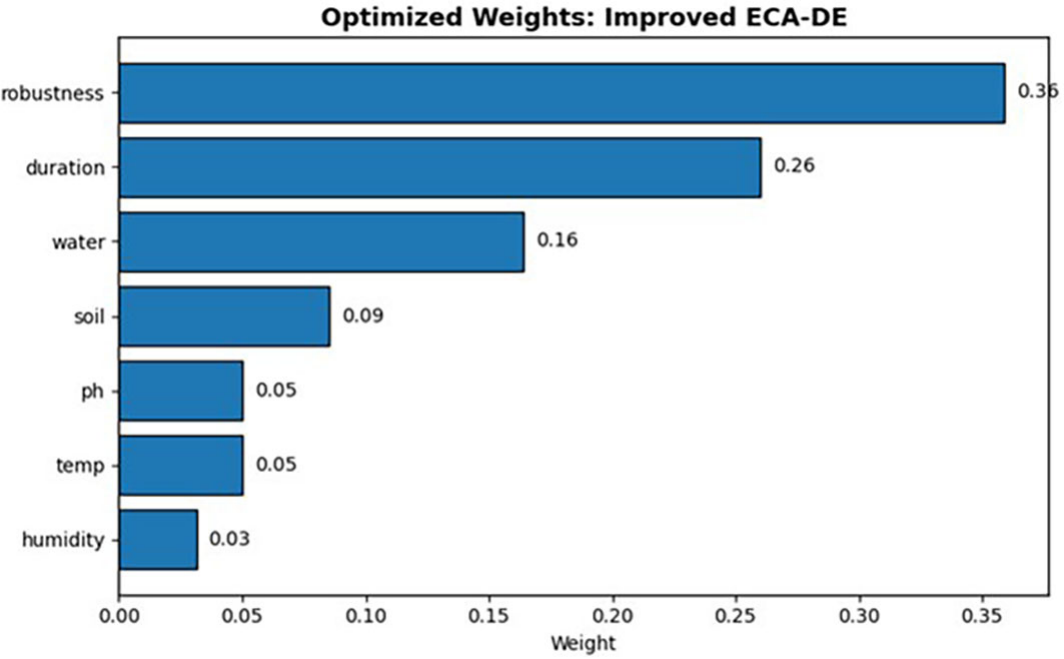
Multi-criteria evaluation of selected crops across key agronomic factors.

**Figure 9:**
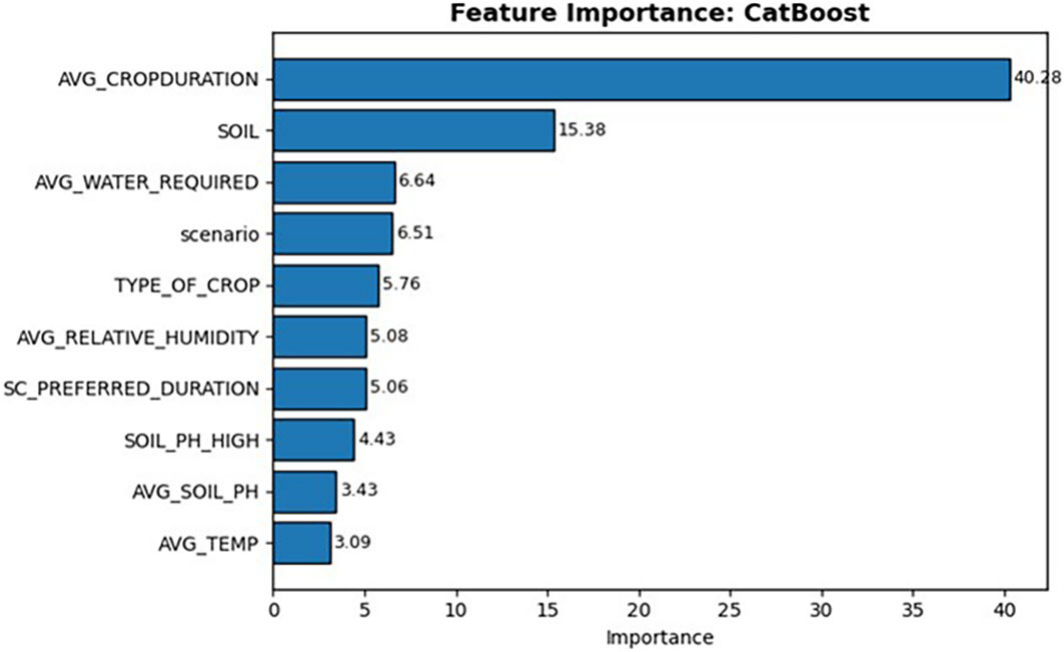
Optimized criterion weights obtained using improved ECA-DE.

**Figure 10:**
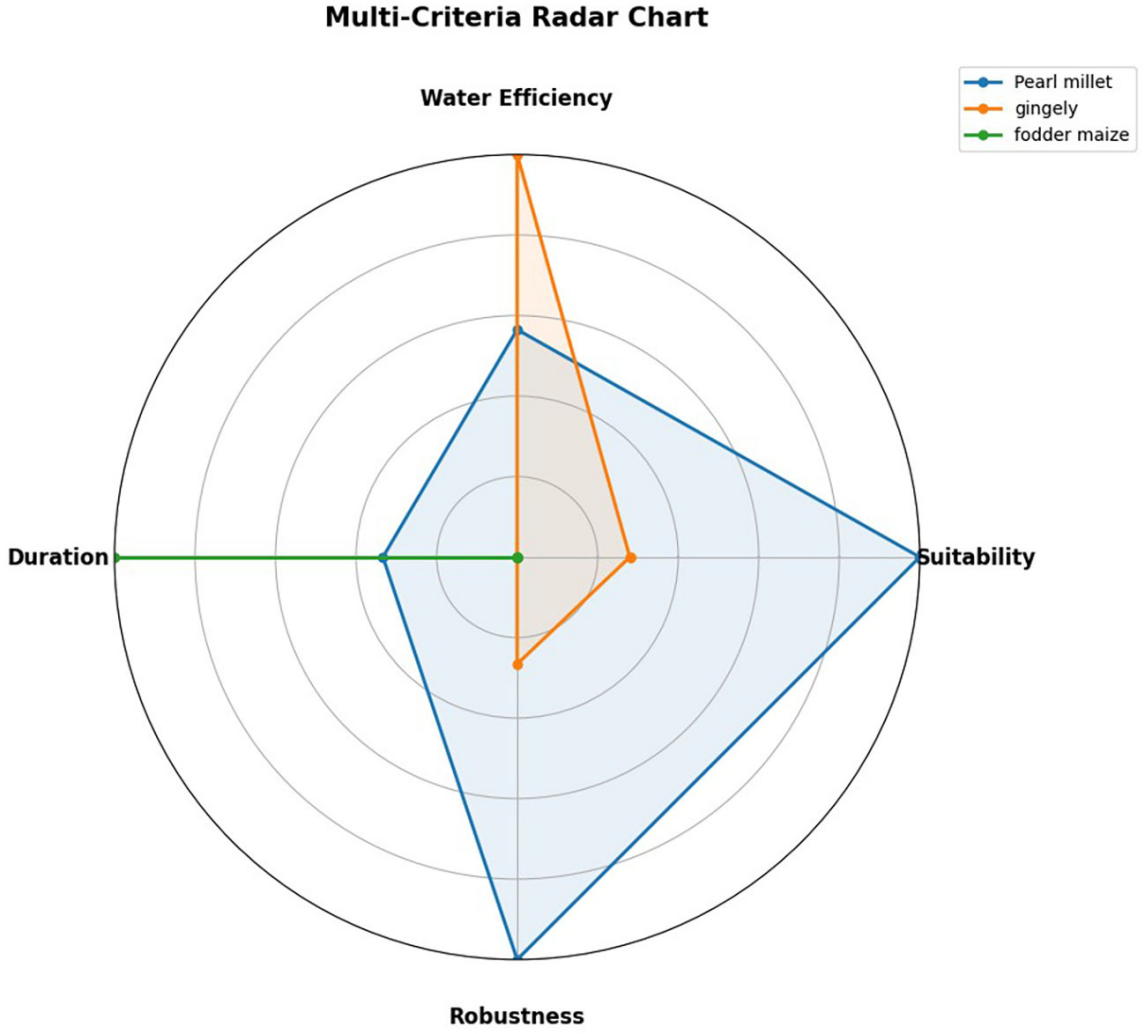
Feature importance analysis of the CatBoost surrogate model.

The case study analysis of the Ariyalur district with equal weightage for the suggested features is presented in **Figures 7**, **8**, **9**, **10** and **Table 5**, which shows the validation of the proposed framework performance. From **Figure 7**, it can be observed that pearl millet (0.75), and gingely (0.72) and fodder maize (0.71) are the top three crops, which are reasonably discriminative in this study. **Figure 8** shows the multi-criteria radar chart, which indicates that pearl millet Has a quite uniform performance in all four criteria, that is, suitability, water efficiency, duration, and robustness, while gingely has much better water efficiency, indicating that it can be cultivated under water-limited conditions. These findings are the results of different agronomic criteria trade-offs were considered while developing the scenario-aware crop recommendation model. The robust model analysis is summarized in **Figure 9**, where the robustness criterion has a maximum weight of 0.36, followed by a duration of 0.26 and with 0.16 weightage on water based on the model. In **Figure 10**, the importance of features in the learning process shows that crop duration and soil, followed by water requirement and scenario-related features, are the most important features in predicting the crop types.

**Table 5:** Top crop recommendations for Ariyalur under balanced scenario.

Rank	Crop	Type	Suitability score	Water Req. (mm)	Duration (days)
1	Pearl millet	Millets	0.753	425	80
2	Gingely	Oil seeds	0.720	375	88
3	Fodder maize	Forage crops	0.707	490	65
4	Fodder cumbu	Forage crops	0.713	490	82
5	Velimasal	Forage crops	0.586	325	50

**Table 5** lists the leading crop recommendations and further corroborates these results. Pearl millet was at the top, with a suitability score of 0.753, requiring a moderate amount of water (425 mm) and having a moderate length (80 days). This was closely followed by gingely and fodder maize. In general, these findings verify that the suggested framework is capable of harmonizing crop yield, resource-saving, and ease of understanding, and hence, can be considered practical for agricultural decision support.

## 5 Conclusion

This study proposed a multifaceted and scenario-sensitive crop recommendation system that includes into six steps: gathering and preparing data, creating scenarios specific to each district, developing an expert suitability score model, Standard DE and Improved ECA-DE based optimization of criteria-weight, CatBoost surrogate training, and constraint-based final ranking and recommendation. The proposed system can accurately reflect the intricate relationships between the environment and agriculture and simultaneously produce crop recommendations that are adaptable to balanced, drought, and short-season scenarios. A major contribution of this study is the integration of scenario-aware crop recommendation, CatBoost-based surrogate learning, and Improved ECA-DE driven multi-criteria optimization within a single district-level agricultural decision-support framework. The CatBoost surrogate model achieved a highly accurate prediction (*R*^2^ = 0. 9975) with a very low error, and the learning curve analysis verified strong generalization without overfitting. A comparison with baseline methods revealed that the proposed approach not only strikes a balance among suitability, water needs, and crop duration, but also considers resource efficiency while ensuring productivity. Even if the Standard DE slightly outperforms the optimization fitness-wise, the Improved ECA-DE is able to deliver almost the same results but with computation time and memory usage that are significantly lower, which makes it a good choice for practical applications. The research conducted in the Ariyalur district proves the framework’s capability to identify the right crops even under natural conditions. In summary, the method proposed here improves the clarity, flexibility, and consistency of decisions, hence it is a fruitful approach in the areas of precision and climate-resilient agriculture. Incorporating real-time weather data, remote sensing data, and explainable AI techniques to improve scalability and transparency will be the future directions. In addition, the present study is primarily centered on agronomic and environmental limitations, such as soil suitability, water availability, climatic adaptation, and crop duration. Economic aspects, such as market demand, cultivation costs, and profitability, were not considered in this study and will be explored in subsequent studies to enhance the real-world use of the suggested scenario-aware crop recommendation system. In addition, real-time weather and remote sensing data for dynamic scenario generation will be included, data-driven suitability scoring mechanisms will be explored to reduce bias, and comparisons with advanced optimization and deep-learning approaches will be performed in the future.

## Data Availability

Publicly available datasets were analyzed in this study. This data can be found here: https://data.mendeley.com/datasets/zyyb98msjc/1.
